# A Carbon-Fiber Sheet Resistor for MR-, CT-, SPECT-, and PET-Compatible Temperature Maintenance in Small Animals

**DOI:** 10.18383/j.tom.2019.00008

**Published:** 2019-06

**Authors:** Veerle Kersemans, Stuart Gilchrist, Sheena Wallington, Philip D. Allen, Ana L. Gomes, Gemma M. Dias, Bart Cornelissen, Paul Kinchesh, Sean C. Smart

**Affiliations:** Department of Oncology, CRUK/MRC Oxford Institute for Radiation Oncology, University of Oxford, Oxford, UK

**Keywords:** carbon fiber, heater, multimodal imaging, MR-CT-PET-SPECT compatibility

## Abstract

A magnetic resonance (MR)-, computed tomography (CT)-, single-photon emission computed tomography (SPECT)-, and positron emission tomography (PET)-compatible carbon-fiber sheet resistor for temperature maintenance in small animals where space limitations prevent the use of circulating fluids was developed. A 250 Ω carbon-fiber sheet resistor was mounted to the underside of an imaging cradle. Alternating current, operating at 99 kHz, and with a power of 1-2 W, was applied to the resistor providing a cradle base temperature of ∼37°C. Temperature control was implemented with a proportional–integral–derivative controller, and temperature maintenance was demonstrated in 4 mice positioned in both MR and PET/SPECT/CT scanners. MR and CT compatibility were also shown, and multimodal MR-CT-PET-SPECT imaging of the mouse abdomen was performed in vivo. Core temperature was maintained at 35.5°C ± 0.2°C. No line-shape, frequency, or image distortions attributable to the current flow through the heater were observed on MR. Upon CT imaging, no heater-related artifacts were observed when carbon-fiber was used. Multimodal imaging was performed and images could be easily coregistered, displayed, analyzed, and presented. Carbon fiber sheet resistors powered with high-frequency alternating current allow homeothermic maintenance that is compatible with multimodal imaging. The heater is small, and it is easy to produce and integrate into multimodal imaging cradles.

## Introduction

In vivo imaging using positron emission tomography (PET), single-photon emission computed tomography (SPECT), and x-ray computed tomography (CT) is increasingly applied in conjunction with magnetic resonance imaging (MRI). In some cases, these systems are colocated within a common gantry system such that the animal is moved between modalities under scanner control. In contrast, where this is not possible, it is advantageous to hold the animal in a cradle that can be moved with the animal in situ such that the animal position within the cradle is constant and image registration is straightforward (1–3). Regardless of the scanner-specific cradle arrangements, it is essential that animals are actively provided with heat to avoid hypothermia that is a consequence of anesthesia (4, 5). It is also essential that the method used for providing thermoregulation is compatible with all of the imaging systems being used, and that it can operate within the physical and technical constraints of each system. MRI scanners, in particular, frequently operate with limited space owing to the loading requirements of radiofrequency (RF) coils (6) and this hinders the use of the most widely used heating systems that include heat lamps, circulating warmed fluids and air, prewarmed heat reservoirs, and exothermic chemical reaction pads (4, 5, 7–10). MRI scanners must avoid the use of large volumes of metal and even small amounts of ferromagnetic materials, while CT scanners, and by inference PET and SPECT scanners where CT-derived intensity corrections are made, must avoid materials of high atomic number in order to avoid image corruptions derived from severe x-ray attenuation (11). MRI must also avoid the formation of magnetic field inhomogeneities consequent to the delivery of electric current.

Compact magnetic resonance (MR)-compatible heaters for homeothermic maintenance in small animals have recently been described (7, 12). In one design, a resistive electrical heater was formed from a tightly-wound twisted pair wire, interfaced to a homeothermic maintenance controller. This heater can be implemented very easily on existing electrical heater systems, and it provided adequate heating performance without causing any significant distortion of the magnetic field or the images. Unfortunately, it is not robustly deformable, and any usage-related change in shape, resulting from normal wear and tear, can lead to imperfect spatial cancellation of stray magnetic field. An alternative heater element was formed from a narrow-gauge wire connected to a high-frequency (99 kHz) alternating current (AC) power source. This frequency was chosen because its cycle time, 10.1 μs, was short enough that long-term field inhomogeneities, and consequent MR image distortions, were absent, while any sound resulting from the heater's use in a magnetic field was inaudible, even for small rodents (12). In contrast to the twisted pair wire, the use of temporal, rather than spatial, averaging of stray magnetic fields toward zero allowed the use of arbitrarily shaped resistors that could be deformable.

While both of these heater elements are compatible with MR, the presence of copper (atomic number, z = 29) leads to excessive x-ray attenuation and streak artifacts in CT images. This obscures anatomy and pathology, and it corrupts the CT-based attenuation maps used for quantification of PET and SPECT images. As such, these types of electrical heaters cannot be used with CT unless they can be formed from an electrically conductive, low-atomic-number element, such as lithium, beryllium, or carbon, as these do not cause attenuation-related image corruptions. Lithium is highly reactive in air; beryllium is very expensive and toxic; while carbon, in the form of resin-embedded carbon-fiber is chemically inert to air, cheap, nontoxic, and easy to work, and is widely used as a heating element in a range of applications. Here, we present a high-frequency AC-based heating resistor in which the metal wire has been replaced by a custom-shaped carbon-fiber sheet to effect a planar heater element that is both MR- and CT-compatible and which can, as a result, be used with CT-corrected PET and SPECT imaging. Improved stability of temperature control was achieved using a proportional–integral–derivative (PID) device that reduced the output power as the temperature approached its target. This is crucial to minimize temperature fluctuations, especially as the MRI system itself delivers an unspecified and variable amount of heat to the animal during operation.

In this report, we describe the use of a carbon-fiber heater, show its multimodal imaging compatibility, and show the generation of a multimodal image of the mouse abdomen formed using dynamic contrast-enhanced MRI (DCE-MRI), CT, ^99m^Tc SPECT, and ^18^F (PET)SPECT; (PET)SPECT describes the SPECT detection of 511-keV photons as used in conventional PET imaging and allows true simultaneous imaging of PET and SPECT isotopes.

## Materials and Methods

### Ethics Statement

All animal studies were performed in full compliance with national legislation and with the approval of the Oxford University Animal Welfare and Ethical Review Body.

### Imaging Cradle and Carbon Fiber Sheet-Resistive Heater Assembly

**Figure 1.**
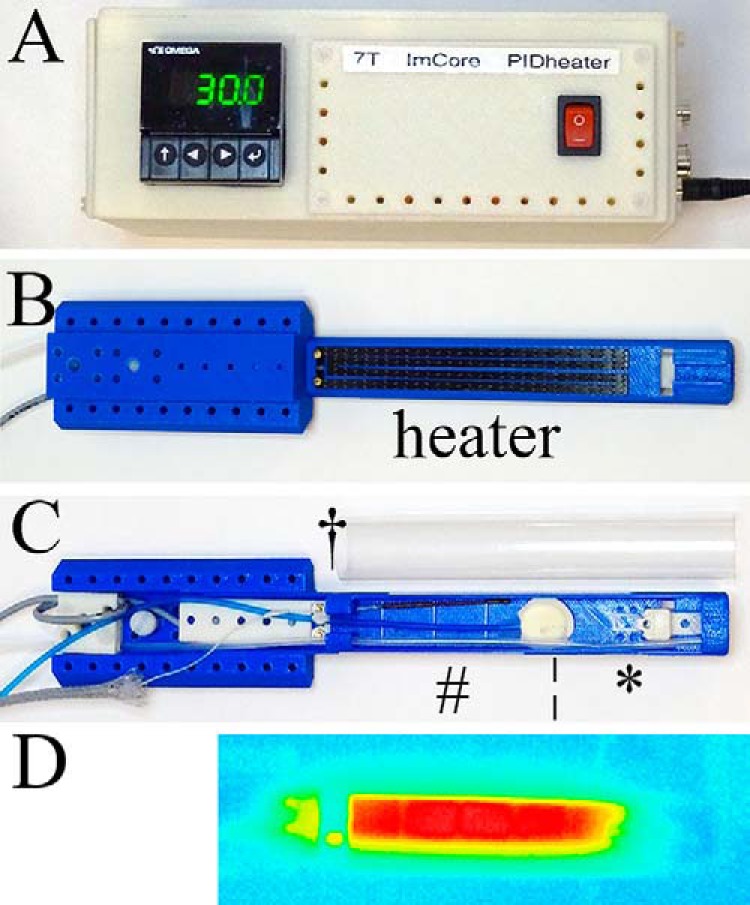
Carbon fiber sheet–resistive heater embedded in a 3D-printed, flat-base multimodal imaging cradle. Proportional–integral–derivative (PID) controller (A); bottom view of the cradle (B); top view of the cradle (C); and thermal image of the cradle surface (D). The cradle contains a heater and (*) a mouthpiece assembly consisting of a base block, a vertical and horizontal adjustable mouth bar, and an anesthetic gas delivery tube, (¦) is a pressure balloon for respiration monitoring, (#) is an optical fiber for temperature monitoring, and (†) is a cover sheet to contain anesthetic gas. The current wires and metal couplings to the carbon-fiber are located beyond the imaging field of view (FOV) so do not present image distortions.

A flat-based MR- and CT-compatible animal support cradle was designed using computer-aided design (CAD) software (SolidWorks, Dassault Systèmes, Hammersmith, UK) and printed using acrylonitrile butadiene styrene (ABS) plastic, on a 3D printer (HP Designjet 3D, Bracknell, UK). A 250-Ω resistive heater element formed from a 0.75-mm-thick carbon-fiber sheet (764-8700, RS Components, Corby, UK) which was cut into 4- × 3-mm-wide legs, each 120-mm in length and 1 mm apart, and glued to the underside of the cradles as shown in Figure 1B. As such, the heater element was positioned 1.68 mm below the lowest part of the object to be imaged.

The heater volume was ∼1.5 mL, occupying 3.05% of the volume of the small-diameter RF coil used for the validation studies reported in this work. The cradle was assembled with an anesthetic gas supply tube, a heater power cable, a fiber optic rectal temperature probe, and a pneumatic respiration monitor (Figure 1C). All services were supplied using “quick-fit” connectors so that the cradle could be disconnected from one imaging system and reconnected on the other quickly and without any risk of the animals recovering from the anesthetic depth used before disconnection.

### AC Heating Control Units

Core body temperature was measured using an optical rectal temperature probe (OTP-M-X-62F2.5-1.5(PTFE/PVC)-XN-7GT-M1-PV0014a, Opsens Solutions, Québec, Canada) connected via a 10-m fiber-optic extension to an AccuSens 4-channel Signal Conditioner (ACS-P4-N-62SC Opsens Solutions, Québec, Canada).

Temperature control was driven using a PID controller (CN16PT-305-DC, OMEGA Engineering Ltd., Manchester, UK; Figure 1A). This controller takes the analog output of the AccuSens unit as the temperature input signal, providing gain control signal to an AC power circuit, which comprises a Pierce oscillator operating at 99-kHz output and a power amplifier (LM1875, Texas Instruments, Dallas, Texas). The maximum output power was limited to ∼2 W for the heating elements described, although this could be increased or decreased for other heating elements. The maximum temperature of the cradle surface was 37.0°C, when a maximum output gain was used indefinitely, and this was demonstrated using a thermal imaging camera (Testo 875-1i, RS Components, Corby, UK). This temperature was selected to ensure thermal burns could not result from the use of this heating apparatus. All animal service signal and power leads entering and leaving the magnet room were passed through RF filters (series 700 high-performance filtered connector, 4000 pF capacitance Pi filter type, part number SCI 56-715-005, API Technology Corp., Milton Keynes, UK). These animal services all operate at low frequencies (<101 kHz) and can pass through the filters, while higher frequencies, some of which could affect MR image quality, cannot.

### Animal Preparation

Female 6- to 8-wk-old CBA/Crl mice (Charles-River, Harlow, UK) were housed in individual ventilated cages at constant temperature and humidity, and water and food were freely available. Anesthesia was induced and maintained using isoflurane (1%–4%) in air/oxygen (v/v 80/20); the animals recovered afterwards with no ill effect. Induction and recovery were performed in a heated unit with a base temperature of ∼37°C. Rectal temperature was set at 36.0°C using the aforementioned heater driver. The depth of anesthesia was monitored using a pressure balloon system measuring the animal's respiration rate, which was maintained at 60–90 breaths/minute. Respiratory signals were processed for use in a gating control of the scanner to allow for respiratory synchronized MRI.

### Assessment of Homeothermic Maintenance in Mice

For validation of thermal stability, mice (n = 4 for MRI; n = 4 for SPECT/(PET)SPECT/CT) were anaesthetized and placed on the imaging cradle in the MRI or SPECT/(PET)SPECT/CT scanner. Rectal temperature was monitored for 50 minutes in total after which the animals recovered.

For MRI, the additional heat load of high-duty cycle MRI scanning was included after the animals had reached their equilibrium temperature.

### Assessment of MRI Compatibility

MR compatibility of the heater element was tested in a water gel phantom (Hydrogel, 70-01-5022, ClearH2O) by using single-shot Point-RESolved Spectroscopy (PRESS) spectroscopy and 2D fast low angle shot (FLASH) imaging, and in vivo in the mouse using respiratory-gated 2D FLASH and cardiorespiratory-gated 3D FLASH imaging. In all cases, the same acquisition was repeated for the heater turned on and off.

MRI was performed on a 7 T, 210-mm VNMRS horizontal bore preclinical imaging system equipped with a 120-mm bore gradient insert (Varian Inc., Palo Alto, California). RF transmission and reception were performed using a 26-mm-diameter, 100-mm-long RF coil for system validation purposes, and a 32-mm-diameter, 45-mm-long RF coil for multimodal imaging (Rapid Biomedical GmbH, Rimpar, Germany).

Single-shot PRESS spectra from a 2 × 2 × 2 mm^3^ voxel positioned at the bottom edge of the water gel phantom, closest to the heater (≈1.68 mm from the heater), were acquired with echo time (TE) = 15 milliseconds, bandwidth = 4 kHz, and complex points = 2048. Raw data were zero-filled to give a real spectrum of 8192 points with 2-Hz exponential line broadening. The full width at half maximum (FWHM) linewidth of this voxel was ∼20 Hz. Spectra were acquired with the heater turned off and on. In addition, a difference spectrum for the heater turned on and off was compared with a spectrum acquired with the heater off. Ten repetitions of a multi-slice spoiled gradient echo scan were acquired on this gel phantom: repetition time (TR) = 100 milliseconds, TE = 10 milliseconds, thickness (THK) = 1 mm, field of view (FOV) = 100 × 25 mm^2^, matrix = 384 × 96, flip angle (FA) = 30°. Dummy scanning was performed for 50 seconds before data acquisition to stabilize a steady state. The total scan time was 146 seconds for 10 repetitions, with each repetition alternating between the heater turned off and on.

In vivo, a multi-slice, constant TR, spoiled gradient echo with respiration-gating and reacquisition (13) was performed: TR = 1000 milliseconds, TE = 6 milliseconds, THK = 1 mm, FOV = 100 × 25 mm^2^, matrix = 512 × 128, FA = 30°. The total scan time was 195 seconds. ImageJ was used to calculate the difference image between the heater turned off and on (14). Ten repetitions of a cardiorespiratory-gated 3D FLASH scan with reacquisition (15) were acquired, with each repetition alternating between the heater turned off and on, and 10 further repeats were acquired with the heater just turned on: TR = 3 milliseconds, TE = 1.28 milliseconds, FOV = 108 × 27 × 27 mm^3^, matrix = 512 × 128 × 128, gradient spoiling with 167 mT/m for 0.85 milliseconds in all three axes, RF hard pulse duration = 16 μs, FA = 5°, and RF spoiling. The total scan time was <5 minutes for the 20 repetitions. Dummy scanning was performed for at least 20 seconds before data acquisition to stabilize a steady state. Image intensity as a function of the repetition number was determined in ImageJ, using a square ROI positioned proximal to the heater.

For multimodal imaging, respiratory-gated 3D FLASH imaging was performed repeatedly to effect DCE-MRI.

Signal-to-noise ratio (SNR) was tested using measurements of the pulse power required to generate a FA excitation pulse of 90°, and by acquisition of a nongated 3D FLASH scan (as per the DCE scan described above), using a postmortem animal (so as not to be affected by the cardiorespiratory motion) and in the presence and absence of the carbon-fiber heating element.

### Assessment of CT Compatibility

CT compatibility was tested in vivo in the absence of any heater and in the presence of either a copper wire or a carbon-fiber sheet heater element. Whole-body CT was performed using the VECTor^4^CT system (MILabs, Utrecht, The Netherlands). Images were acquired at 50 kV and 0.24 mA using continuous rotation (40°/s) and were reconstructed using a cone-beam filtered backprojection (Feldkamp algorithm) on a 0.2-mm voxel grid. Beam hardening and ring artifacts were corrected, and the voxel numbers were converted into Hounsfield units by using a premeasured calibration factor.

### Demonstration of Multimodal (PET)SPECT/SPECT/CT/MR Imaging

Multimodal MR-CT-(PET)SPECT-SPECT imaging of the abdomen, including the kidneys, was performed on animals that were held in the same cradle as for MRI and using anesthesia and physiological maintenance as described above.

(PET)SPECT and SPECT imaging was performed using the single-gantry VECTor^4^CT system fitted with the HE-UHR-RM PET/SPECT collimator (1.8-mm pinholes); (PET)SPECT describes the SPECT detection of 511-keV photons as used in conventional PET imaging. CT, SPECT, and (PET)SPECT imaging were performed immediately before MRI; ^111^In-citrate and ^18^F-fluorodeoxyglucose (FDG) were used for SPECT and (PET)SPECT imaging, respectively. DCE was performed (scan details in Assessment of MRI Compatibility) with a scan time of ∼10–15 s/frame. Contrast agent (100 μL, Gadospin, Viscover, Germany) was infused in the lateral tail vein over 17 seconds at the start of frame 11/50.

Data were acquired in list mode (0–1200 keV) using MILabs acquisition software v7.39. Triple energy window–based scatter correction was applied for each photon peak window (^18^F: 460-562 keV with background windows set to 439.6–460 keV and 562–582.4 keV; ^111^In: 155.7–190.3 keV and 222.3–271.7 keV with background windows set to 148.8-155.7 keV, 190.3–197.2 keV, 212.4–222.3 keV, and 271.7–281.6 keV). All images were reconstructed with MILabs reconstruction software v3.24 on 0.8-mm isotropic 3D voxel grids using dual matrix similarity regulated ordered-subset expectation maximization (dual matrix SROSEM) (8). After reconstruction, the (PET)SPECT, SPECT, and their corresponding CT data were coregistered and resampled to equivalent 200-μm voxel sizes. CT-based attenuation correction was applied. Reconstructed images were viewed and analyzed using PMOD v.3.37 (PMOD Technologies, Zurich, Switzerland). ^111^In-citrate was produced as follows: 50 MBq of ^111^InCl_3_ (Curium, UK) was incubated with 0.05M sodium citrate (Sigma-Aldrich, UK) in a total volume of 0.17 mL at 37°C for 1 h; pH was adjusted with 1M NaOH. ^111^In-citrate (SPECT; 10.95 MBq) and ^18^F-fluorodeoxyglucose (FDG, (PET)SPECT; 10.55 MBq) were coinjected through a cannula into the lateral tail vein, and data were acquired for 12 minutes (2 bed positions containing both kidneys; 21 frames of 35 seconds; injection during frame, 2/20, lasting for 2 frames; 0–1200 keV). Frames 16–21 were summed and reconstructed into 1 imaged (4 iterations). A CT image (scan details in Assessment of CT Compatibility) was acquired for anatomical referencing and to aid coregistration between CT, SPECT, (PET)SPECT, and DCE-MRI. Following CT, SPECT, and (PET)SPECT imaging, the cradle containing the mouse in situ was transferred to MRI for DCE-MRI (scan details in Assessment of MRI Compatibility). MR and CT images were aligned and overlaid using rigid-body registration. The resulting transformation matrix was used to overlay the SPECT and (PET)SPECT images onto the MR and CT images.

Further MR-CT-(PET)SPECT imaging has been performed using this heating system in several hundred mice undergoing study for other purposes.

## Results

### Homeothermic Maintenance

Ambient room and magnet bore temperature was 19.3°C and 20.4°C, respectively, as measured with the fiber optic temperature probe.

**Figure 2.**
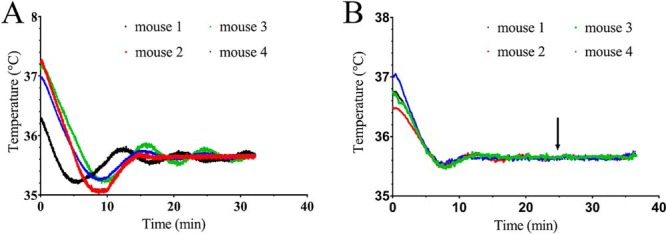
Homeothermic maintenance of core temperature in CBA mice using a carbon-fiber sheet resistor powered with high-frequency AC under PID control. The target rectal temperature was set to 36.0°C. Core temperature of 4 mice placed into the SPECT/(PET)SPECT/CT scanner (A). Core temperature of 4 mice placed into the MRI scanner (B). The arrow indicates when respiratory-gated balanced steady-state free-precession (bssfp) imaging was initiated to replicate the additional heat load of high-duty-cycle magnetic resonance imaging (MRI) scanning.

For a set target temperature of 36.0°C, the core temperature equilibrated within 20 minutes and was maintained at 35.5°C ± 0.2°C in 8 CBA mice (Figure 2); the animals recovered quickly and without incident. The animal's initial heat loss upon induction of anesthesia recovered within 10 and 15 minutes for SPECT/(PET)SPECT/CT (Figure 2A) and MRI (Figure 2B), respectively. A stable core temperature of 35.6°C ± 0.1°C was reached for MRI when an additional heat load of high-duty-cycle scanning was applied (Figure 2B).

As the animals approached the target temperature of 36.0°C, the current delivered through the resistor was reduced by the PID to avoid any overheating. The PID also limited the maximum temperature of the upper surface of the cradle to ∼37°C, even when the temperature controller is functioning continually at its maximum output. In addition, the thermal imaging camera showed a homogenous heat distribution along the cradle surface (Figure 1D).

### MRI Compatibility

**Figure 3.**
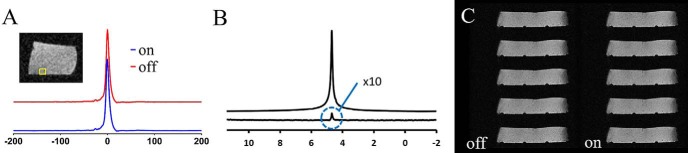
Impact of current flow through the carbon-fiber sheet heater element on MRI of a water gel phantom. PRESS pulse-acquire spectroscopy with the heater turned off and on (A). The image shows the placement of the 2-mm-cubic PRESS voxel that was located 3, 7, and 5 mm off-isocentre in the *z*, *y*, and *x* axes, respectively, such that it was positioned as close to one of the heater legs as possible; absolute value display of the spectrum showing the water resonance with the heater turned off (top trace) and the difference spectrum from acquisitions made with and without current flow through the heater (bottom trace) (B). The intensity of the difference spectrum is scaled 10× higher than the top trace and the absolute value display was used for clarity of display of the residual subtraction error. T1-weighted 2D FLASH MRI on a water gel phantom with the heater turned off (left column) and on (right column) (C).

**Figure 4.**
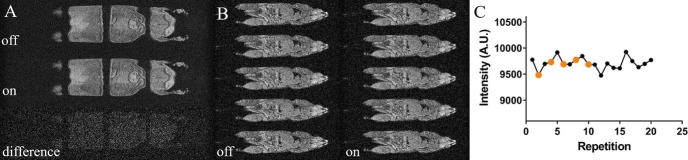
Impact of current flow through the carbon-fiber sheet heater element on in vivo MRI of the mouse. T1-weighted in vivo whole-body respiration-gated 2D FLASH MRI with the heater turned off (top) and on (middle) (A). The bottom image displays the difference image between both images. Cardiorespiratory-gated whole-body 3D FLASH MRI with the heater turned off (left column) and on (right column) (B). The average intensity over a square, placed close to the heater surface, was plotted for 20 repetitions with the heater turned on (black dots) or off (orange dots) (C).

Spectral and imaging distortions resulting from the use of a current flow through the heater elements were not seen (Figures 3 and 4). The MR spectrum acquired from a PRESS voxel in a water gel phantom showed no line–shape distortions or frequency shifts attributable to the current flow through the heater (Figure 3A). A difference spectrum acquired with the heater turned on and off was compared with the spectrum acquired with the heater turned off; both are shown in Figure 3B. The latter spectrum is displayed in the absolute value mode, and the difference spectrum was magnified 10× to render the difference spectrum clearly. The spectra show that the current flowing through the heater has a negligible effect on the proton resonance frequency at distances exceeding 1.68 mm, the thickness of the cradle. We have not further tested the minimum operating distance at which distortions could occur. Similarly, no distortions attributable to the current were observed in the same water gel phantom in 2D FLASH imaging (Figure 3B), with the difference image showing negligible residual image intensity. In vivo imaging using respiratory-gated 2D FLASH (Figure 4A) and cardiorespiratory-gated 3D FLASH (Figure 4B) confirmed the gel phantom data as again no distortions attributable to the current flow were observed. In all cases, no differences were observed when the heater was turned off and turned on, which was further emphasized when the signal intensity was plotted against scan repetition (Figure 4C).

For a postmortem mouse with the heater pad in position, a 1.5-dB increase in reference pulse power was required. SNR for 5 × 5 voxel regions of interest positioned in the liver (centrally in the coil) and at the extreme edge of the z-FOV was reduced by ∼20%, approximately in line with the increase in reference pulse power, as described elsewhere (6) (SNR = 12.52 and 10.25 for the absence and presence of the heater, respectively). The loss in SNR was a direct consequence of loading interactions with the tune/match circuits as the variable ranges of some of the tuning and matching capacitors were operated at their extremes. The size of the effect is dependent upon the exact position of the heater within the coil and its tune/match circuits and upon the size of both heater and coil.

### CT Compatibility

**Figure 5.**
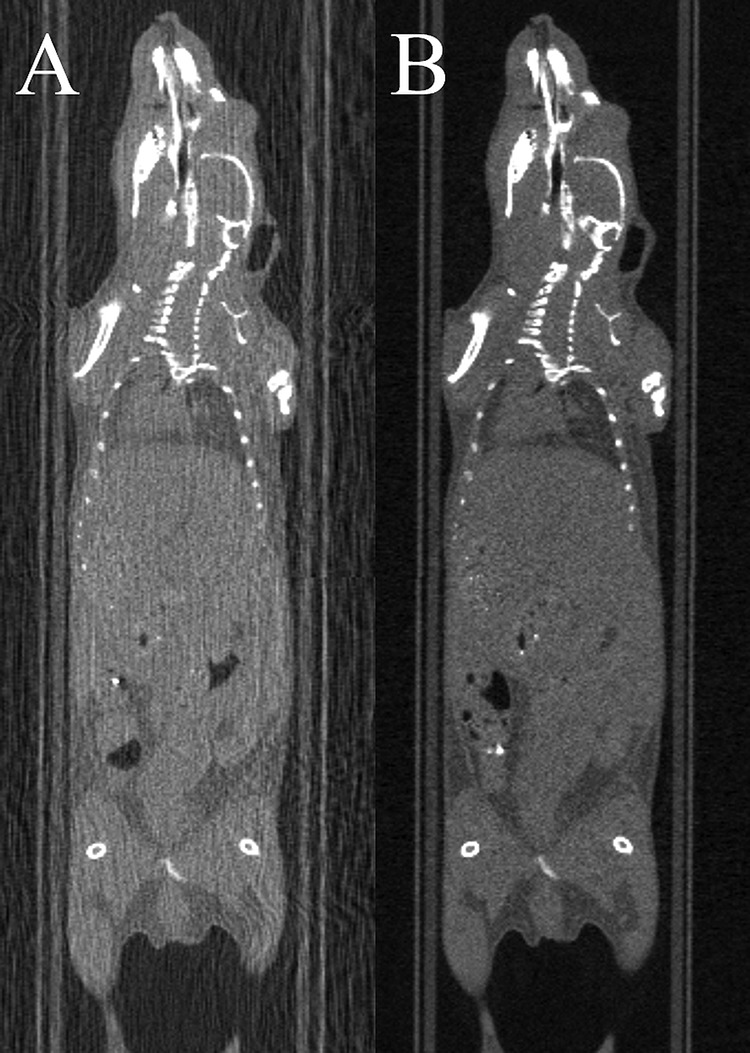
Impact of the heater material on computed tomography (CT) image quality. CT imaging using the 150-µm-diameter copper wire heater (A) or the carbon-fiber sheet heater (B). Streak artifacts owing to the presence of the heater are absent when using the carbon-fiber heater, and the image intensities remain intact.

Imaging distortions resulting from the presence of the carbon-fiber heater element were not seen (Figure 5B). The presence of the copper wire heater element produced severe image streaking (Figure 5A), organs were poorly defined, and CT intensities were poorly estimated.

### Multimodal PET/SPECT/CT Imaging and MRI Imaging

**Figure 6.**
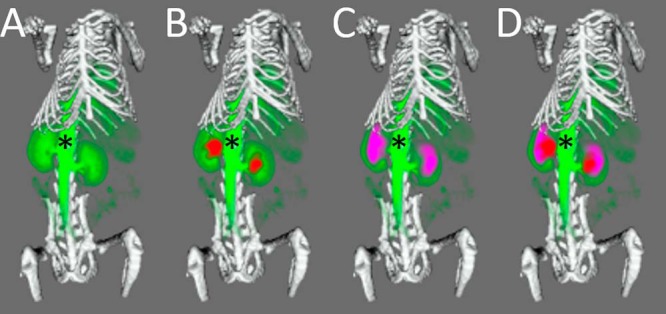
Multimodal imaging of a mouse using the carbon-fiber sheet–resistive heater embedded in a 3D-printed, flat-base, multimodal imaging cradle. The skeleton, kidneys, and major vessels to the kidneys (*) are marked up. The skeleton (white) was imaged by CT, while ^111^In-citrate (red), ^18^F-fluorodeoxyglucose (purple), and gadodiamide (green) were used for SPECT, (PET)SPECT, and DCE-MRI of the kidneys, respectively. Each panel shows an additional layer of the coregistered, segmented image: CT + MRI (A), CT + MRI + SPECT (B), CT + MRI + (PET)SPECT (C), CT + MRI + (PET)SPECT + SPECT (D).

A composite image formed from DCE-MRI, CT, ^111^In SPECT, and ^18^F (PET)SPECT is shown in Figure 6. The CT and MR images were used to drive the rigid-body registration.

Multimodal (PET)SPECT/SPECT/CT/DCE-MRI imaging was performed, and images could be easily coregistered, displayed, analyzed, and presented (Figure 6). The kidneys as rendered from the MRI image overlaid well with those rendered from the CT, (PET)SPECT, and SPECT images.

## Discussion

The power dissipation of the carbon-fiber sheet heater allowed homeothermic maintenance of the animals. The maximum power delivery of ∼2 W provided a maximum cradle temperature of ∼37°C, thus avoiding any possibility of burns, and all animals recovered without incident. Animals entered the study with temperatures in the range of 36.3°–37.3°, presumably as a result of a different activity level ranges of the mice, local environment stability, and exact positioning of the rectal thermometer tip within the body. The homeothermic maintenance was performed using a custom-made PID-based temperature controller in conjunction with a standard commercial thermometer. This resulted in automated temperature maintenance for MR, (PET)SPECT/SPECT, and CT applications with a fluctuation of 0.1°C at ∼0.5°C below the target temperature. Given that this heating system is used in imaging scanners that are both actively cooled and heated simultaneously, we accepted that this 0.5°C PID undershoot error was tolerable, especially as the temperatures were stable. The heat delivery, assessed with a thermal imaging camera (Figure 1D), was homogeneous when compared with that achieved with our previous versions of wire heater elements for which the wires ran markedly hotter than the average temperature of the heater pads (thermal images of the wire resistors not shown). Homeothermic control of the core temperature was shown, even in the presence of additional heat input from the scanner. The latter scan-induced heating can also be used to reduce the time necessary for the animal to recover from its initial heat loss following anesthetic induction.

MR compatibility of the AC-driven carbon-fiber heater was shown in localized spectroscopy and FLASH imaging (Figures 3 and 4). The equivalence of spectra and images acquired in the presence and absence of AC heating shows that the heater element delivers heat in a manner that does not corrupt the imaging process, and the carbon-fiber material does not lead to any marked image distortions.

CT-compatibility was shown in Figure 5. The absence of attenuation-derived streak artifacts provides good image quality that enables quantitative analysis of image intensities. This was not the case where the copper wire heater was used for which significantly streak artefacts were observed. As such, the carbon-fiber sheet heater performed favorably when compared with the copper wire heater.

Integration of the carbon-fiber heater into the imaging cradle was straightforward, and the low space requirement of the heater allowed the imaging to be performed in the smallest RF coil appropriate for whole-body imaging. The carbon-fiber heating element reduced SNR by ∼15%–20%, equating to the use of an ∼30-mm, rather than the 26-mm, diameter coil assuming that SNR is proportional to 1/coil volume (6). While the use of larger RF coils may allow temperature maintenance using circulating fluids in MRI, they are not necessarily an option in space-limited, small-bore collimators for PET and SPECT imaging, or when direct access to the animal is required. In such cases, the heating system described provides an elegant solution.

Moreover, by using CAD software in conjunction with a 3D plastic printer, it is very simple to design and deploy imaging cradles that feature integrated carbon-fiber heater elements. In our facility, we have produced many replicates of the heater element, which are then integrated into several different shapes and sizes of cradles, each of which has identical spaces and mounting points for the heater elements. The cradles also have integrated locating adaptors so that they can be easily positioned on scanners supplied by different manufacturers, each with their own bespoke mounting systems. Having performed the scans, the undistorted MR and CT images could be registered using a simple rigid-body registration. As the CT, SPECT, and (PET)SPECT images were generated within the same system, it was straightforward to overlay the SPECT and (PET)SPECT images onto the MR images and produce the composite image shown in Figure 6. This image shows, to a first approximation, blood volume from DCE-MRI, glucose distribution, and renal excretion to the bladder from FDG (PET)SPECT, renal excretion to the bladder of ^111^In-citrate from SPECT, and anatomical definition of the skeleton from CT.

In conclusion, carbon-fiber sheet resistors powered with high-frequency AC allow homeothermic maintenance that is compatible with multimodality imaging using MR, CT, PET, and SPECT. The heater is small, and easy to produce, and integrate into cradles for multimodal imaging.
